# Evaluation of a Multidisciplinary Antimicrobial Stewardship Program in a Saudi Critical Care Unit: A Quasi-Experimental Study

**DOI:** 10.3389/fphar.2020.570238

**Published:** 2021-03-10

**Authors:** Abdul Haseeb, Hani Saleh Faidah, Manal Al-Gethamy, Muhammad Shahid Iqbal, Abrar Mohammed Barnawi, Shuruq S. Elahe, Duha Nabeel Bukhari, Turki Mohammad Noor Al-Sulaimani, Mohammad Fadaaq, Saad Alghamdi, Waleed Hassan Almalki, Zikria Saleem, Mahmoud Essam Elrggal, Amer Hayat Khan, Mohammed A Algarni, Sami S. Ashgar, Mohamed Azmi Hassali

**Affiliations:** ^1^ Department of Clinical Pharmacy, College of Pharmacy, Umm Al-Qura University, Al-Abdia Campus, Makkah, Saudi Arabia; ^2^ Department of Microbiology, Faculty of Medicine, Umm Al Qura University, Saudi Arabia; ^3^ Department of Infection Prevention and Control Program, Alnoor Specialist Hospital Makkah, Makkah, Kingdom of Saudi Arabia; ^4^ Department of Clinical Pharmacy, College of Pharmacy, Prince Sattam Bin Abdulaziz University, Al-Kharj, Saudi Arabia; ^5^ King Faisal Specialist Hospital and Research Centre, Jeddah, Kingdom of Saudi Arabia; ^6^ Skaggs School of Pharmacy and Pharmaceutical Sciences, University of California San Diego, San Diego, United States; ^7^ Department of Pharmacy, International Medical Center, Jeddah, Kingdom of Saudi Arabia; ^8^ Ajyad Emergency Hospital, Ministry of Health, Makkah, Saudi Arabia; ^9^ Laboratory Medicine Department, Faculty of Applied Medical Sciences, Umm Al Qura University, Makkah, Saudi Arabia; ^10^ Department of Toxicology and Pharmacology, College of Pharmacy, Umm Al Qura University, Makkah, Saudi Arabia; ^11^ Department of Pharmacy Practice, Faculty of Pharmacy, The University of Lahore, New Campus, Lahore, Pakistan; ^12^ Clinical Pharmacy Department, School of Pharmaceutical Sciences, Universiti Science Malaysia, Penang, Malaysia; ^13^ Microbiology Laboratory, King Abdulaziz University Hospital, Jeddah, Saudi Arabia; ^14^ Assistant Professor of Medical Microbiology, College of Medicine, Umm Al Qura University, Makkah, Saudi Arabia; ^15^ Discipline of Social and Administrative Pharmacy, School of Pharmaceutical Sciences, Universiti Sains Malaysia, Penang, Malaysia

**Keywords:** antimicrobial stewardship, multidisciplinary approach, critical care, Saudi Arabia (KSA), quasi experimental approach

## Abstract

**Background:** Antimicrobial stewardship programs (ASPs) are collaborative efforts to optimize antimicrobial use in healthcare institutions through evidence-based quality improvement strategies. With regard to critically ill patients, appropriate antimicrobial usage is of significance, and any delay in therapy increases their risk of mortality. Therefore, the implementation of structured multidisciplinary ASPs in critical care settings is of the utmost importance to promote the judicious use of antimicrobials.

**Methods:** This quasi-experimental study evaluating a multidisciplinary ASP in a 20-bed critical care setting was conducted from January 1, 2016 to July 31, 2017. Outcomes were compared nine months before and after ASP implementation. The national antimicrobial stewardship toolkit by Ministry of health was reviewed and the hospital antibiotic prescribing policy was accordingly modified. The antimicrobial stewardship algorithm (Start Smart and Then Focus) and an ASP toolkit were distributed to all intensive care unit staff. Prospective audit and feedback, in addition to prescribing forms for common infectious diseases and education, were the primary antimicrobial strategies.

**Results:** We found that the mean total monthly antimicrobial consumption measured as defined daily dose per 100 bed days was reduced by 25% (742.86 vs. 555.33; *p* = 0.110) compared to 7% in the control condition (tracer medications) (35.35 vs. 38.10; *p* = 0.735). Interestingly, there was a negative impact on cost in the post-intervention phase. Interestingly, the use of intravenous ceftriaxone measured as defined daily dose per 100 bed days was decreased by 82% (94.32 vs. 16.68; *p* = 0.008), whereas oral levofloxacin use was increased by 84% (26.75 vs. 172.29; *p* = 0.008) in the intensive care unit.

**Conclusion:** Overall, involvement of higher administration in multidisciplinary ASP committees, daily audit and feedback by clinical pharmacists and physicians with infectious disease training, continuous educational activities about antimicrobial use and resistance, use of local antimicrobial prescribing guidelines based on up-to-date antibiogram, and support from the intensive care team can optimize antibiotic use in Saudi healthcare institutions.

## Introduction

Owing to critically ill patients’ unstable hemodynamic status, antimicrobial therapy for the management of severe infections is challenging. Antimicrobial selection, in particular, proves difficult as every delay increases patients’ mortality risk. In critical care settings, antimicrobial selection involves numerous problems, the most significant among which are suitability in terms of disease pathophysiology and the need to consider patient characteristics, such as renal and hepatic function, hemodynamic status, and risk of adverse drug reactions. Therefore, inappropriate antimicrobial use increases the chances of adverse drug reactions, *Clostridioides difficile* infections, and multidrug-resistant pathogen development (Brusselaers et al., 2011; Taggart et al., 2015). In this context, antimicrobial stewardship strategies are the only evidence-based solution as per the recommendations of global health regulatory agencies.

A systematic review by Kaki et al. (2011) demonstrated that antimicrobial stewardship programs (ASPs) in intensive care units (ICUs) can be successful through the use of institution-specific strategies. The majority of these studies focused on the impact of ASPs on antimicrobial consumption and cost. However, for ASPs in critical care settings to be effective, in addition to ensuring adherence to institutional goals and strategies, they must promote prompt and rational antibiotic use for life-threatening infections. An ICU ASP may exhibit some very ICU-specific goals and strategies (Brusselaers et al., 2011; Kaki et al., 2011). According to the Centers for disease Control and Prevention (CDC), ASPs in healthcare institutions must be multidisciplinary, encompassing involvement of leadership, clinical pharmacy, microbiology, and clinician education and training regarding infectious disease state management, recent antimicrobial use and trends, and optimal prescribing strategies (Pollack and Srinivasan, 2014).

As there is little margin for error with regard to ICU patients, the early initiation of broad-spectrum antimicrobial therapy to cover all possible pathogens and subsequent targeting of infective pathogens based on clinical and microbiological findings has been indicated (Prowle et al., 2011). Based on this concept, according to the National Health Services strategy (Start Smart and Then Focus) for the implementation of successful ASPs in secondary care, it is important to emphasize the initiation of effective antimicrobial therapy in the first hour of admission among critically ill patients and review the decision in 48 h. Antimicrobial therapy is then either to be continued or stopped through intravenous (IV) to oral conversion, de-escalation to narrow-spectrum agents, or switching to outpatient parenteral antibiotic therapy (Ashiru-Oredope et al., 2012, Supplementary Material). In addition, clinical decision support systems are vital in acute care settings to ensure appropriate and timely antimicrobial delivery for life-threatening infectious conditions (Lawrence and Kollef, 2009).

In Saudi Arabia, the concept of antimicrobial stewardship has been gaining ground since the 2014 introduction of the implementation process by the Ministry of Health’s General Administration of Pharmaceutical Care at public sector hospitals. According to their strategic plan, as of 2017, the ASP implementation process was in stage 4. One of the barriers to successful ASP implementation in the Saudi context is the lack of human resources (Alomi, 2017). To date, there have been limited Saudi studies evaluating the effectiveness of multidisciplinary ASPs in critical care settings (Amer et al., 2013; AlAwdah et al., 2015). Therefore, we recently introduced a multidisciplinary ASP in a critical care setting by adopting the CDC’s core antimicrobial stewardship elements and the National Health Services’ Start Smart and then Focus strategy. The national ASP toolkit was modified according to the hospital population and local resistance pattern in Makkah city. A prominent factor in the program was the involvement of higher administration at the leadership level and the use of education as a supplementary strategy to utilize current human resources. We used quasi experimental study to evaluate the impact of multidisciplinary antimicrobial stewardship interventions on antimicrobial use with cost and clinical outcomes as the primary and secondary outcome, respectively.

## Material and Methods

### Design

**FIGURE 1:**
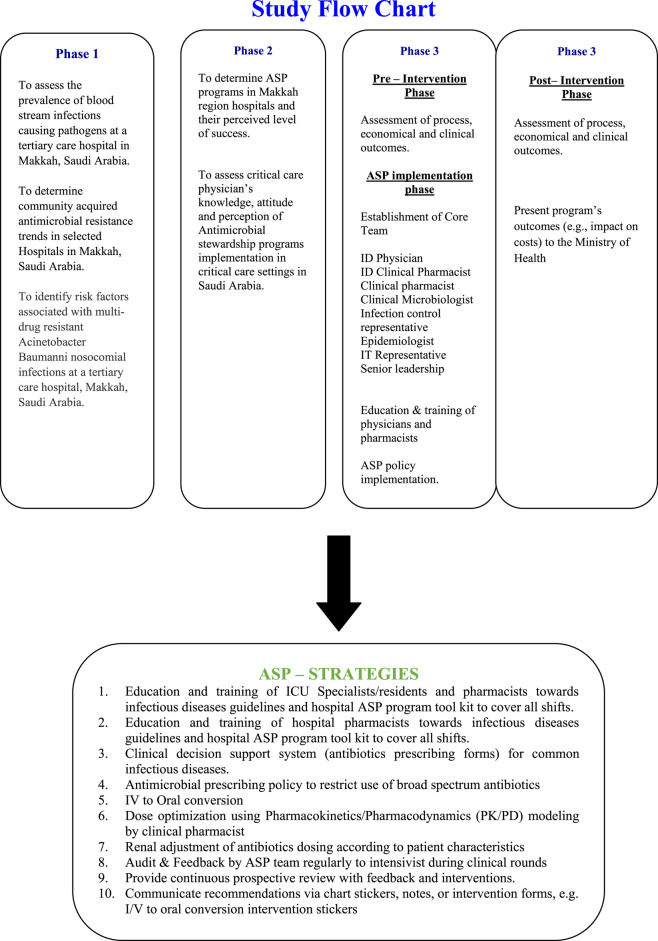
Study flow chart.

This study, conducted from January 1, 2016 to July 31, 2017, employed a quasi-experimental design with nine months of pre-intervention and post-intervention data, respectively. Detail of study phases is provided in Figure 1.

### Setting and Population

The setting was a 20-bed adult ICU at Ajyad Emergency Hospital, a secondary care hospital in Makkah city. On a yearly average, more than 5000 pilgrims and Makkah residents receive acute medical care at this hospital. Antimicrobial use and other outcomes were measured with a special drug utilization review form for all admitted patients. As the hospital only provides inpatient services in ICU settings, all ICU patients were included in the study.

All prescribers from the institution were included in the knowledge assessment survey and continuous medical education (CME) sessions. As per hospital policy, antimicrobials are only prescribed for admitted patients. Antibiotic use and other outcomes were collected for all patients admitted to the ICU during the study period.

### Outcomes

#### Primary and Secondary Outcomes

The primary outcome was total systemic antimicrobial consumption in the ICU calculated as defined daily dose (DDD) per 100 bed days per month. Antimicrobial use was calculated as total grams per month from patient charts for inpatient use and pharmacy dispensing records. Patient admission records were retrieved from the medical record database. The total cost per 100 bed days per month for each antimicrobial and classes, measured in Saudi riyals (US $1.00 = 3.74 SR). Antimicrobial agent cost was retrieved from the Saudi Drug Formulary published by the Saudi Food and Drug Authority. All antimicrobial costs are publicly available online on the Saudi Food and Drug Authority site.

Secondary outcomes were Clinical outcomes included all-cause mortality, infectious disease-related mortality, and mean ICU length of stay. In addition, patient demographics, including gender, age, nationality, comorbidities, and final diagnosis, were recorded.

#### Control/Tracer Medications

As the primary goal of the ASP was a decline in antimicrobial consumption, medications used for the prophylaxis of stress-induced ulcer were used as the control condition. Therefore, H2 blockers and proton pump inhibitors, measured in DDD per 100 bed days, were used as negative tracer medications, since prescription of these agents was unrelated to the ASP intervention.

#### Programmatic Outcomes (Post-intervention Only)

The rates of conformance to the ASP team’s interventions were recorded to gain an understanding of the ICU team’s acceptance of these measures. These post-intervention data were only retrieved after ASP implementation.

### Antimicrobial Stewardship Implementation Process

First, a multidisciplinary antimicrobial stewardship subcommittee was developed under the leadership of the hospital director to facilitate stewardship protocol implementation. The committee represents leadership from all disciplines of hospital, including critical care, emergency, clinical microbiology, infection control, quality and pharmaceutical care departments.

Second, the National Antimicrobial Stewardship toolkit by Ministry of Health (Alomi, 2017) was reviewed and the hospital’s antibiotic prescription policy was accordingly modified. The antimicrobial stewardship algorithm (Start Smart and then focus) was distributed to all ICU staff in addition to the ASP toolkit (Ashiru-Oredope et al., 2012, Supplementary Material).

#### Recommended Strategies

A multidisciplinary antimicrobial stewardship strategies were implemented with focus on education and training of ICU specialists/residents and pharmacists regarding infectious disease guidelines and use of the hospital ASP toolkit in all shifts. Also, antimicrobial prescribing forms adopted from the recent national ASP toolkit were implemented by incorporating IV to oral conversion, renal dose adjustment and dose optimization strategies targeting pharmacokinetic/pharmacodynamic therapeutic targets. Regular audit and feedback to the intensivist by the multidisciplinary ASP team was delivered during clinical rounds.

The pre-intervention and post intervention period were defined as January 1, 2016 to September 30, 2016 and November 1, 2016 to July 31, 2017, respectively. During the pre-intervention period, the antimicrobial stewardship committee was created and the study outcomes were retrospectively developed from patients’ medical records. As per hospital regulations, medical records for the past two years were available for all patients; thus, there were no missing data in the retrospective review.

During the post-intervention period, the multidisciplinary ASP team reviewed all patients admitted to the ICU on a daily basis. Patient charts were regularly reviewed until discharged. Verbal ASP recommendations were made during clinical rounds; these were subsequently documented in a specific ASP follow-up form in patients’ medical charts. Intensivists had full authority to accept or decline intervention based on patients’ clinical condition. This strategy ensured the ICU team’s prescribing autonomy.

#### Physician and Pharmacist Training and CME

According to a statement from the General Administration of Pharmaceutical Care, one of the barriers to the implementation of ASP in healthcare institutions is the lack of personnel. Therefore, to utilize existing human resources for efficient ASP implementation, we decided to provide educational sessions about infectious diseases to prescribers and pharmacists. Education interventions are key to antimicrobial stewardship implementation and have been recommended by the World Health Organization (WHO) (Cooke et al., 2010; Pulcini and Gyssens, 2013).

A CME program was arranged for all prescribers, pharmacists and nursing staff in the hospital. It included special sessions regarding awareness of hospital-wide antimicrobial use guidelines and restriction strategies toward restricted antimicrobials. In addition, special educational sessions were designed to educate on basic microbiology, antimicrobial pharmacology, and pharmacokinetics and pharmacodynamics as well as hospital guidelines regarding acute respiratory tract infection management in adults. These educational sessions were used to supplement antimicrobial stewardship activity. There were two sessions per day, three days a week during the intervention period. Each session lasted 1 h. During the intervention phase, the sessions covered awareness regarding hospital antibiotic prescribing policies, predesigned prescribing forms as clinical decision support, IV to oral conversion strategies, dose optimization, and renal dosing.

#### General Antimicrobial Stewardship Sessions

These sessions were conducted just before the pre-intervention phase and focused on general microbiology, pharmacology, and infection control issues in practice. This helped enhance trainees’ knowledge about antimicrobial selection for specific organisms. Members of the ASP committee and experts in clinical microbiology and infectious diseases from local hospitals conducted didactic sessions on clinical microbiology, clinical pharmacology, the importance of antimicrobial stewardship, and the significance of core strategies to promote clinical outcomes throughout the institution.

#### Targeted Antimicrobial Stewardship Educational Sessions

These sessions focused on antimicrobial prescribing guidelines and awareness of the hospital’s ASP. There was a special focus on prescribing forms for common infectious diseases. Pharmacists and physicians specializing in infectious diseases conducted these sessions.

### Statistical Analysis

All statistical analysis were performed with SPSS version 22 (IBM Corp., Armonk, NY, United States). Simple descriptive statistics were used to present the sample’s demographic characteristics. For the analysis of the primary outcome measure, we used the WHO-recommended statistical measure to calculate drug consumption: DDD per 100 bed days as monthly consumption data. Categorical variables were assessed using the chi-square test or Fisher’s exact test and continuous variables were assessed using the *t*-test or Man Whitney *U* test. *p*-values less than 0.05 were considered statistically significant.

### Ethical Approval

This study was approved by the Directorate General of Health, Ministry of Health, Makkah region with reference number 47/300/43,149 based on ethical approval from the Al-Noor Specialist Hospital Ethics Review Board. The research ethics board waived the need for informed consent as the study used anonymized patient data.

## Results

During the pre-intervention phase, 684 patients were admitted to the ICU, among whom 135 (19.7%) had confirmed infectious diseases. Similarly, in the post-intervention period, there were 623 patients admitted to the ICU, among whom 169 (27.1%) had confirmed infectious diseases. There were no significant differences in gender, age, nationality, and final diagnosis between the pre- and post-intervention periods (Table 1). There were few differences in comorbidities between the two groups.

**TABLE 1:** Demographic characteristics of study population.

Demographic characteristics	Pre- intervention n (%)	Post- intervention n (%)
**Admissions**		
Total patients admitted in ICU^a^	684	623
Total patients with confirmed infectious diseases	135 (19.7)	169 (27.1)
**Gender**		
Male	60 (44.5)	70 (41.5)
Female	75 (55.5)	99 (58.5)
**Age (mean, SD)**	56 (14)	58 (12)
**Nationality**		
Southern Asia	78 (58)	91 (53.8)
South Eastern Asia	22 (16.2)	32 (19)
Western Asia	15 (11.1)	23 (13.6)
Western Africa	10 (7.5)	12 (7.1)
Northern Africa	4 (3)	6 (3.5)
Western Europe	6 (4.4)	5 (3)
**Patients status**		
Umrah pilgrims	108 (80)	163 (96.4)
Hajj pilgrims	27 (20)	6 (3.6)
**Final diagnosis**		
Mild/Moderate community acquired pneumonia	71 (52.5)	93 (55)
Severe community acquired pneumonia	45 (33.3)	56 (33.1)
Ventilator associated pneumonia/Nosocomial	3 (2.2)	5 (2.9)
Bronchitis	15 (11.1)	15 (8.8)
Sepsis/Septic shock^b^	20 (14.8)	22 (13)
**Co-morbidities** ^c^		
Hypertension	34 (25.1)	35 (20.7)
Heart failure	11 (8.1)	15 (8.8)
Coronary Artery diseases	27 (20)	21 (12.4)
Diabetes mellitus	47 (34.8)	61 (36)
Asthma	80 (59.2)	96 (56.8)
Chronic obstructive pulmonary disease	20 (14.8)	26 (15.3)
Chronic renal failure	22 (16.2)	27 (15.9)

^a^ intensive care units (ICU).

^b^ Patients. With severe community acquired pneumonia developed septic shock.

^c^ Patients were with more than one Comorbidities.

The mean total monthly antimicrobial utilization decreased by 25% from 742.86 DDD per 100 bed days to 555.33 DDD per 100 bed days after ASP implementation (*p* = 0.110). There was a significant decrease in total IV antimicrobial usage by 129 DDD per 100 bed days after the intervention (*p* = 0.038). There was no significant change (*p* = 0.347) in oral antimicrobial usage in the post-intervention period. With respect to specific antimicrobials, there was a significant drop in ceftriaxone use; there was a mean reduction of 77.64 (*p* = 0.008) DDD per 100 bed days. The mean total monthly consumption of oral levofloxacin was significantly increased by 145.54 DDD per 100 bed days from 26.75 DDD per 100 bed days to 172.29 DDD per 100 bed days (*p* = 0.008). There were no significant changes (*p* = 0.767) in the use of H2 blockers or proton pump inhibitors in the ICU between the two study phases (Table 2).

**TABLE 2:** Comparison of the total monthly consumption of antimicrobials measured as defined daily dose per 100 bed days in the pre and post-intervention phases.

Antimicrobials	Pre-intervention Mean ± S.D	Post-intervention Mean±	*p* value^b^	Maximum estimation of mean reduction
Amoxicillin + clavulanic Acid (I.V)^a^	5.34 ± 4.8	4.11 ± 4.7	0.753	1.23
Amoxicillin + clavulanic Acid (*p*.0)^a^	20.92 ± 24.9	15.06 ± 12.2	0.953	5.86
Piperacillin + tazobactam (I.V)	19.15 ± 12.2	6.47 ± 6.2	0.015	12.75
Cefuroxime (P.O)	234.55 ± 259.7	49.67 ± 33.6	0.036	184.88
Cefuroxime (I.V)	9.51 ± 3.8	1.10 ± 3.3	0.011	8.41
Ceftriaxone (I.V)	94.32 ± 55.6	16.68 ± 8.8	0.008	77.64
Ceftazidime (I.V)	1.20 ± 1.6	1.50 ± 2.5	0.917	0.3
Meropenem (I.V)	8.51 ± 7.1	18.60 ± 24.4	0.441	10.09
Levofloxacin (I.V)	19.02 ± 10.3	55.95 ± 28.5	*0.011*	36.94
Levofloxacin (P.O)	26.75 ± 18.8	172.29 ± 94.8	*0.008*	145.54
Ciprofloxacin (I.V)	12.22 ± 15.9	9.73 ± 9.7	0.735	2.49
Ciprofloxacin (P.O)	3.74 ± 6.2	4.62 ± 12.2	1.000	0.88
Azithromycin (I.V)	20.00 ± 16.2	9.19 ± 12.2	0.161	10.81
Azithromycin (P.O)	16.17 ± 30.0	6.38 ± 13.8	0.686	9.79
Clarithromycin (P.O)	15.37 ± 17.0	9.28 ± 15.1	0.398	6.09
Vancomycin (I.V)	24.73 ± 18.4	8.40 ± 8.5	0.008	16.33
Linezolid (I.V)	0.00 ± 0.0	10.32 ± 8.2	*0.012*	10.32
Amikacin (I.V)	1.34 ± 2.0	0.44 ± 0.9	0.273	0.9
Metronidazole (I.V)	25.61 ± 17.3	16.72 ± 22.3	0.173	8.89
Fluconazole (I.V)	28.40 ± 35.9	6.74 ± 6.3	0.139	21.66
Fluconazole (P.O)	33.88 ± 57.0	3.77 ± 6.0	0.063	30.11
Oseltamivir (P.O)	122.09 ± 61.1	128.32 ± 36.8	0.687	6.23
All antimicrobials	742.86 ± 399.0	555.33 ± 166.7	0.110	187.53
All IV antimicrobials total	293.43 ± 135.1	164.41 ± 68.1	0.038	129.02
All oral antimicrobials total	469.84 ± 310.5	398.67 ± 127.5	0.347	71.17
Total anti PUD^a^ medication (control)	35.35 ± 20.7	38.10 ± 24.28	0.767	2.75

^a^ Intravascular (I.V), Per Oral (P.O), Peptic Ulcer disease (PUD).

^b^ t-test for parametric data and Mann Whitney U for non-parametric data.

There was no significant change in cost as Saudi Riyals (US $1.00 = 3.74 SR) between the periods. The mean total cost of antimicrobials increased from 38,723.72 Saudi riyals per 100 bed days before the intervention to 87,552.70 Saudi Riyals per 100 bed days after the intervention (*p* = 0.678). This non-significant change was due to the inclusion of linezolid in the formulary in the post-intervention phase. There was a significant decline in total mean cost (37%; *p* = 0.008) when the cost of linezolid was not considered in the post-intervention phase. There was a significant change in the mean cost of IV and oral antimicrobials, with a mean reduction of 49,486.77 Saudi Riyals (*p* = 0.021) for IV antimicrobials and 657.8 Saudi Riyals (*p* = 0.021) for oral antimicrobials after ASP implementation (Table 3).

**TABLE 3:** Comparison of the total cost of antimicrobials measured as Saudi Riyals in **cost per 100 bed days in the pre and post-intervention phases.**

Antimicrobials	Pre-Intervention Mean ± S.D	Post-Intervention Mean ± S.D	*p* value^b^	Maximum estimation of reduction
Amoxicillin + clavulanic Acid (I.V)^a^	336.56 ± 303.5	258.81 ± 297.7	0.735	77.75
Amoxicillin + clavulanic Acid (*p*.0)^a^	146.46 ± 174.4	105.45 ± 85.7	0.953	41.01
Piperacillin + tazobactam (I.V)	6837.24 ± 4344.5	2308.23 ± 2214.3	0.015	4529.01
Cefuroxime (P.O)	1817.74 ± 2012.6	384.94 ± 260.7	0.036	1432.8
Cefuroxime (I.V)	413.63 ± 164.4	47.87 ± 143.6	0.011	365.76
Ceftriaxone (I.V)	8,677.83 ± 5112.5	1534.56 ± 807.6	0.008	7143.27
Ceftazidime (I.V)	248.88 ± 334.8	311.60 ± 511.1	0.917	62.72
Meropenem (I.V)	1889.97 ± 1564.4	4130.11 ± 5428.7	0.441	2240.14
Levofloxacin (I.V)	2391.45 ± 1296.4	7035.62 ± 3581.1	*0.011*	4644.17
Levofloxacin (P.O)	427.98 ± 301.0	2756.66 ± 1517.8	*0.008*	2328.68
Ciprofloxacin (I.V)	1939.15 ± 2518.3	1543.57 ± 1536.2	0.735	395.58
Ciprofloxacin (P.O)	52.33 ± 87.1	64.65 ± 136.1	1.000	12.32
Azithromycin (I.V)	1199.81 ± 975.9	551.31 ± 734.9	0.161	648.5
Azithromycin (P.O)	263.81 ± 481.4	102.17 ± 221.6	0.500	161.64
Clarithromycin (P.O)	138.31 ± 152.8	83.51 ± 135.7	0.398	54.8
Vancomycin (I.V)	4600.47 ± 3416.8	1561.72 ± 1572.2	0.008	3038.75
Linezolid (I.V)	0.00 ± 0.0	62,253.46 ± 49,551.3	*0.012*	62,253.46
Amikacin (I.V)	335.55 ± 442.6	95.09 ± 188.8	0.138	240.46
Metronidazole (I.V)	61.90 ± 30.1	35.52 ± 47.3	0.086	26.38
Fluconazole (I.V)	3767.36 ± 4619.7	519.10 ± 487.0	0.051	3248.26
Fluconazole (P.O)	1538.31 ± 2589.0	171.10 ± 271.7	0.063	1367.21
Oseltamivir (P.O)	1638.98 ± 825.0	1697.66 ± 487.7	0.678	58.68
All IV antimicrobials total	32,699.80 ± 15,145.0	82,186.57 ± 54,258.4	0.021	49,486.77
All oral antimicrobials total	6023.93 ± 4126.2	5366.13 ± 1782.1	0.021	657.8
All antimicrobials total	38,723.72 ± 18,033.3	87,552.70 ± 55,719.2	0.678	48,828.98
All antimicrobials (without linezolid)	38,723.72 ± 1833	25,299.24 ± 9815	0.086	13,424.48
Total anti PUD^a^ medications (control)	835.92 ± 724.4	526.02 ± 471.3	0.26	309.9

^a^ Intravascular (I.V), Per Oral (P.O), Peptic Ulcer disease (PUD).

^b^ t-test for parametric data and Mann Whitney U for non-parametric data.

There were no significant changes in infectious disease-related mortality rate, length of stay in the ICU, and proportion of patients admitted and discharged from ICU between the pre- and post-intervention periods (Table 4). The most common ASP recommendation was IV to oral conversion, addition of antibiotics, and de-escalation. Almost all interventions were accepted by physicians, with a few disagreements according to patients’ clinical conditions (Table 5).

**TABLE 4:** Clinical outcomes in the pre and post-intervention phase.

Clinical Outcomes	Pre- Intervention n (%) Total = 135	Post- Intervention n (%) Total = 169	*p*-Value
All-cause mortality^a^	54 (40)	48 (23.6)	0.661
Infectious diseases related mortality ^a^	14 (10.37)	10 (5.9)	0.447
Total mortality (ICU)^a^	68 (50.37)	58 (29.5)	0.501
Clinically cured (discharged)^a^	122 (90.3)	159 (94)	0.338
ICU length of stay (mean)^b^	4.11	4	0.788

^a^ Chi Square test.

^b^ Student t-test.

**TABLE 5:** Detail of interventions recommended by ASP team and their acceptance rate during post intervention phase.

Programmatic Outcomes (Post Intervention Only)	Total (N)	Acceptance Rate
Iv to oral conversion	131	87
Dosage change	76	100
Addition of antibiotics	43	100
Streamlining/De-escalation	28	94
Level check (vancomycin and Aminoglycosides)	9	100

## Discussion

This study marked the adoption of CDC core elements to facilitate the implementation of an ASP in an acute care setting. The multidisciplinary approach resulted in significant outcomes because of the involvement of higher administration. An immediate drop in restricted antimicrobials was observed, with overall decreases in piperacillin + tazobactam (66.5%), ceftriaxone (82.3%), and vancomycin (66%) after ASP implementation. On the contrary, there was a substantial increase in the usage of antibiotics such as meropenem (54%), linezolid (100%), and IV levofloxacin (66%) owing to the timely delivery of appropriate antimicrobials in the context of life-threatening infectious diseases like sepsis and severe community acquired pneumonia. In the pre-intervention phase, ceftriaxone was the drug of choice for most intensivists in the ICU. Similarly, vancomycin was the only anti-methicillin-resistant *Staphylococcus aureus* (MRSA) agent for ICU patients. Linezolid was introduced in the formulary to cover infections with suspected MRSA origin in patients with renal instability.

No significant immediate reductions were observed for control medications, suggesting that the interventions were responsible for change in antimicrobial use. However, there was no appreciable change in total mean cost for antimicrobials in the post-intervention phase, mainly because of the formulary’s introduction of linezolid, the costliest antibiotic among all classes. In addition, meropenem and levofloxacin use also contributed positively to the rise in costs.

Overall, the magnitude of variation in antibiotic usage was similar to the findings reported in a systemic review in critical care settings (Kaki et al., 2011). Similar changes in total antimicrobial use were observed in a Hungarian ICU with restriction policy as the major intervention. Antibiotic consumption decreased by 37.8% (from 162.9 to 101.3 DDD per 100 patients days) (Peto et al., 2008). Similarly, in a Canadian study, the implementation of an audit and feedback strategy in the ICU led to a reduction in mean total antimicrobial use in DDD per 100 bed days by 28% (Taggart et al., 2015). Likewise, in studies in France (Geissler et al., 2003) and Germany (Meyer et al., 2007), similar reductions in total antimicrobial use were observed.

As the main goal of ASP follow-up was to restrict all antimicrobials to the right patients, reductions were mainly observed for restricted antibiotics with compensatory increases in the use of non-restricted antibiotics, for example, levofloxacin for the initial management of community acquired pneumonia. This phenomenon is commonly known as the “squeezing-the-balloon” effect (Burke, 1998). Therefore, the strength of our study lies in the overall reduction of all antimicrobial use, relatively than specific antibiotics only.

As antimicrobial resistance relates to the selective pressure exerted by the overuse of antibiotics (Herwaldt and Duncan, 1997), based on pre-intervention findings, we propose changes in the use of third-generation cephalosporin to improve antibiotic susceptibility and reduce infections related to hospital mortality in critically ill patients (Du et al., 2003). In our study, one of the justifications for the reduced use of third-generation cephalosporin was the implementation of a pneumonia protocol through prescribing forms. According to this form, the first choice in most cases was a high dose of levofloxacin, a respiratory quinolone. In addition, ceftriaxone with azithromycin was not a good choice in cardiac patients because of the risk of azithromycin-induced arrhythmias (Ray et al., 2012; Albert et al., 2014).

Another important finding in our study was the 82.3% reduction in the use of ceftriaxone. This is important as irrational selection of initial antimicrobials could inevitably enhance the chance of mortality in patients with life-threatening infections (Torres et al., 1990; McGowan, 1994). Recent guidelines for the treatment of severe community acquired pneumonia generally recommend levofloxacin (Dunbar et al., 2003; Mandell et al., 2007). Therefore, based on this rationale, the pre-intervention use of ceftriaxone, and the fact that a change in empirical antibiotics may reduce resistance to common enterobacteriaceae-associated infections and improve patient management in the ICU, we focused on replacing ceftriaxone with levofloxacin for empirical therapy for community acquired pneumonia (Mebis et al., 1998). Most intensivists welcomed this strategy and accepted the majority of the ASP team’s recommendations.

Our findings were consistent with a previous result regarding the impact of ASPs on clinical outcomes. In a systematic review by MacDougall and Polk (2005), limiting antibiotic use had no significant impact on an institution’s clinical outcomes (Peto et al., 2008) (MacDougall and Polk, 2005; Peto et al., 2008). Interestingly, no cases of *Clostridium difficile* infection were reported in either the pre- or post-intervention period.

The total increase in mean antimicrobial use achieved through the implementation of the ASP was similar to an ICU study from other Saudi regions. In a study by Amer et al. (2013), ASP interventions caused a reduction in antibiotic use from 2403.64 DDD per 1000 patient days to 376.2 DDD per 1000 patient days. While antimicrobial consumption was lower in our study, our economic outcomes are similar to their findings (Amer et al., 2013; AlAwdah et al., 2015).

In summary, this study supports the use of CDC core elements for the successful implementation of an institutional ASP. Education and training along with the involvement of an ICU team with basic infectious disease training and support from leadership are effective tools for the achievement of antimicrobial stewardship goals.

### Conclusion

Successful ASP implementation has been reported to be useful in increasing compliance to hospital antibiotic prescribing policies and limiting irrational antimicrobial use. Such improvements can indirectly reduce antimicrobial resistance. This is in line with the results of the current study, where a significant reduction in broad-spectrum antimicrobials was observed. However, the increase in cost owing to increased linezolid use raised the issue of reserving it for particular patients for cost-saving purposes.

Overall, it is evident that a multidisciplinary approach and leadership involvement strengthen the implementation process.

#### Limitations

Despite its strengths, this study has certain limitations. First, the results may represent the success of one particular ASP and may not be generalizable to other hospitals in Saudi Arabia. Second, the primary outcome measure was antimicrobial consumption rather than the appropriateness of antimicrobial therapy. Antimicrobial prices were obtained from the Saudi National Formulary and Saudi Food and Drug Authority site as the baseline; therefore, the referenced cost may vary from the Ministry of Health’s purchase cost from companies. We were unable to obtain actual purchase prices owing to regulatory restrictions.

The use of DDDs to measure antimicrobial consumption may be challenging as antibiotic dosing in the ICU varies from patient to patient because of the variable pathophysiology of diseases and patients’ hemodynamic status. In addition, as per the WHO, DDDs are not suitable for all patients because of dosing criteria variation in critically ill patients. Streamlining use to narrow-spectrum antibiotics can increase DDD consumption. There were monthly variations in patient admission rates because of seasonal variations in the Hajj and Umrah pilgrimages.

#### Recommendations for Future Studies

The findings from this study can be used to develop the standard of practice for antimicrobial stewardship implementation in healthcare settings in the Makkah region. The results can encourage new ideas while planning ASPs in other contexts, for example, neonatal ICUs, maternity settings, and so on.

The implementation of admission-level rapid diagnostics to differentiate between viral and bacterial infection should be studied as an ASP intervention. Further, the impact of early initiation of targeted antimicrobial therapy on clinical outcomes should be assessed.

A similar study over an extended period and on a larger scale in a tertiary care setting is recommended. A pre-post intervention spanning two years will address the long-term impact of seasonal variation on antimicrobial consumption.

## Data Availability

The original contributions presented in the study are included in the article/Supplementary Material, further inquiries can be directed to the corresponding author.
