# Supplementary material for: Evaluation of a Multidisciplinary Antimicrobial Stewardship Program in a Saudi Critical Care Unit: A Quasi-Experimental Study
Source: Front Pharmacol. 2021 Mar 10;11:570238. doi: 10.3389/fphar.2020.570238 (PMC7988078; doi:10.3389/fphar.2020.570238)
Supplement: Supplementary file 1 [file datasheet1.docx]

**EVALUATION OF MULTIDISCIPLINARY ANTIMICROBIAL STEWARDSHIP PROGRAMS IN A SAUDI CRITICAL CARE UNIT USING QUASI**

**EXPERIMENTAL STUDY DESIGN**

**Supplementary Material**


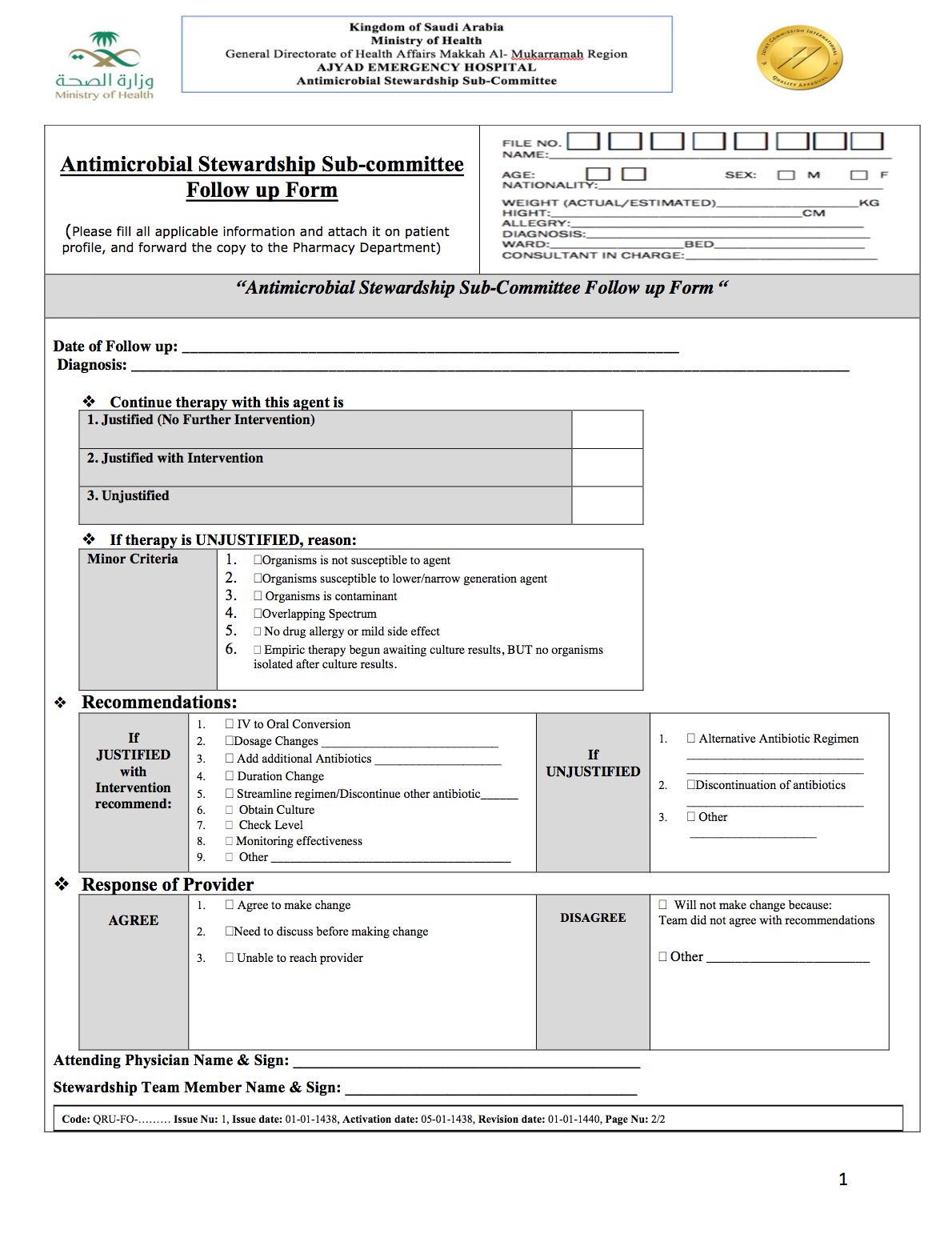


**
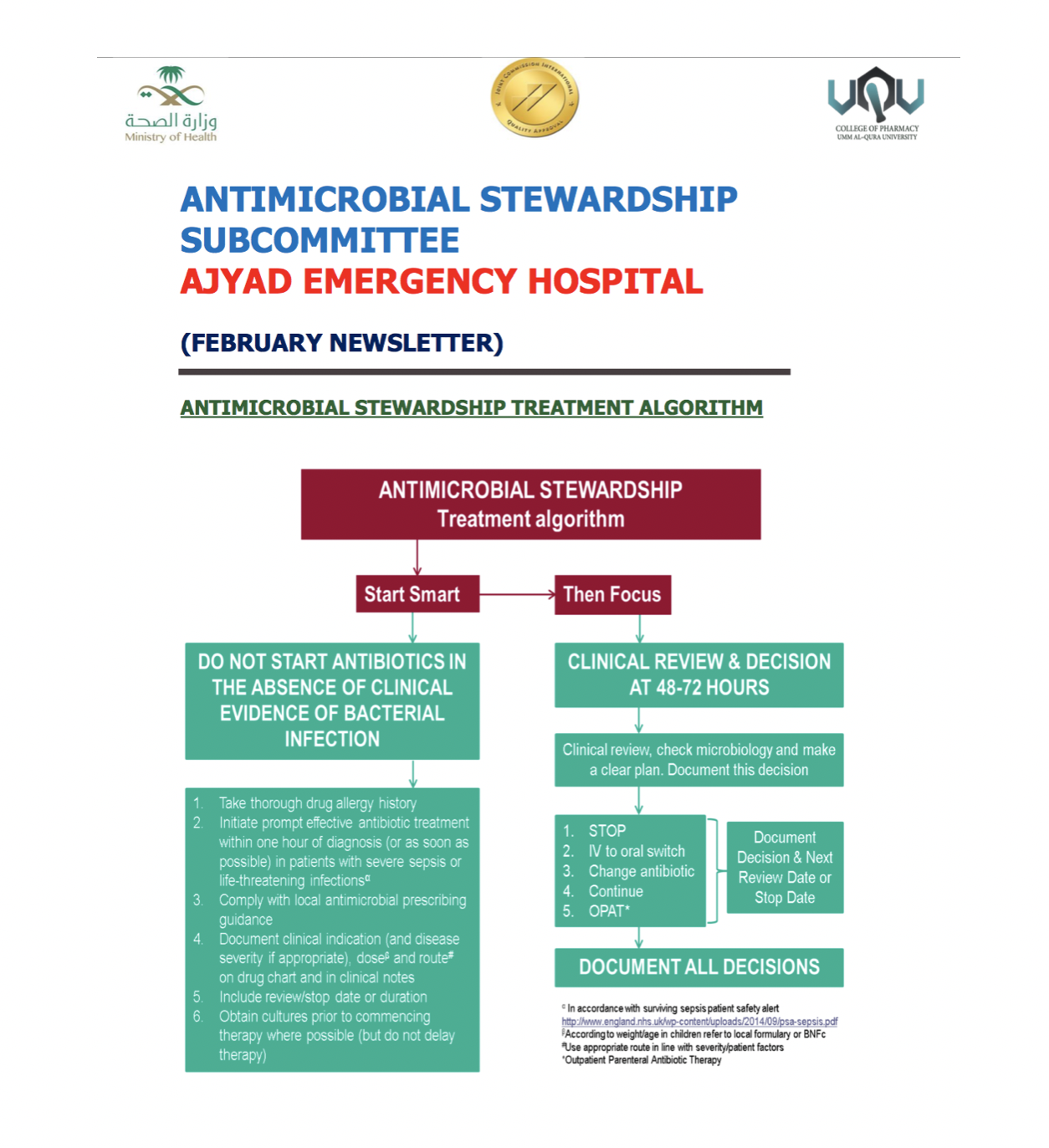
**

**
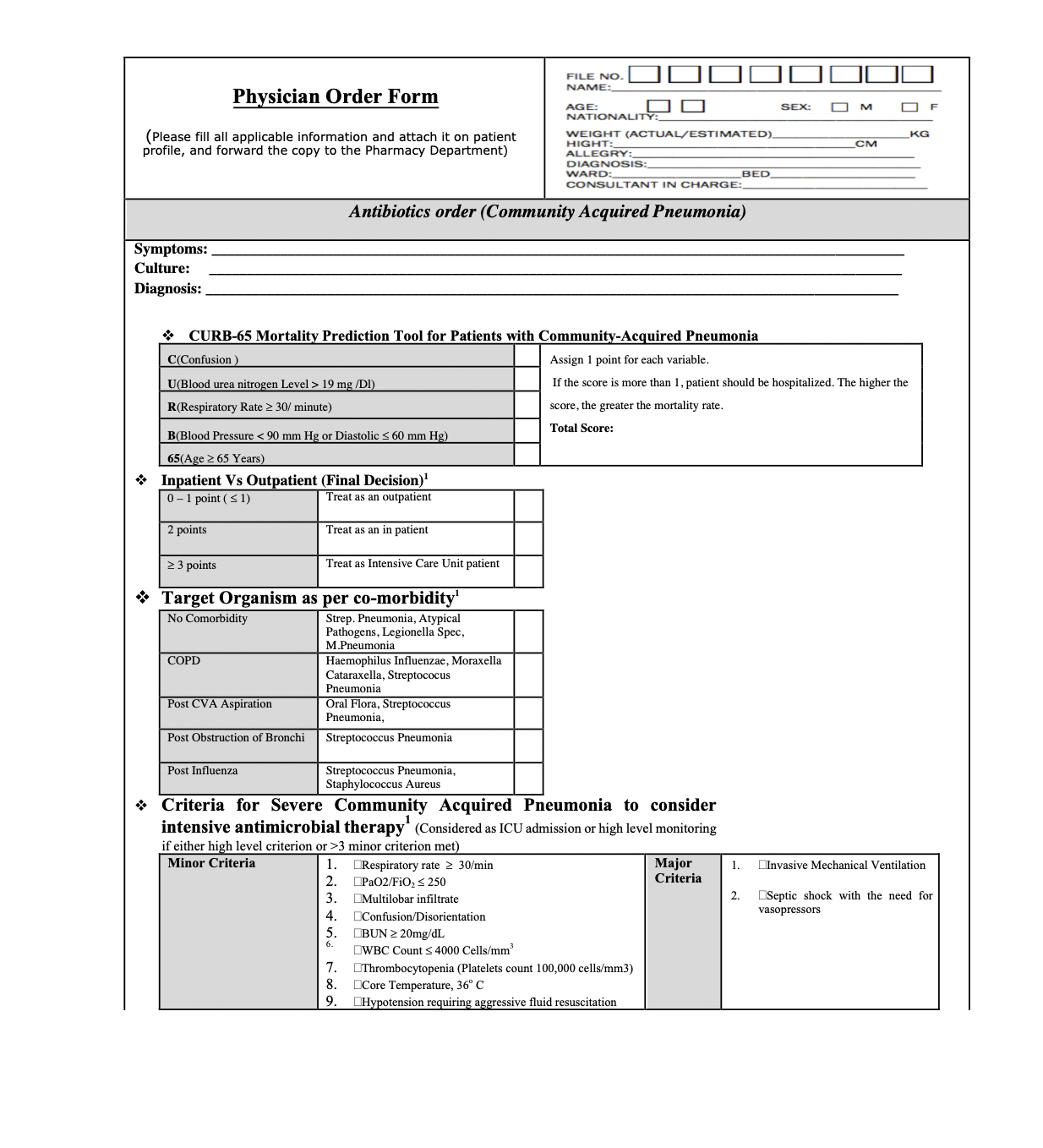
**

**
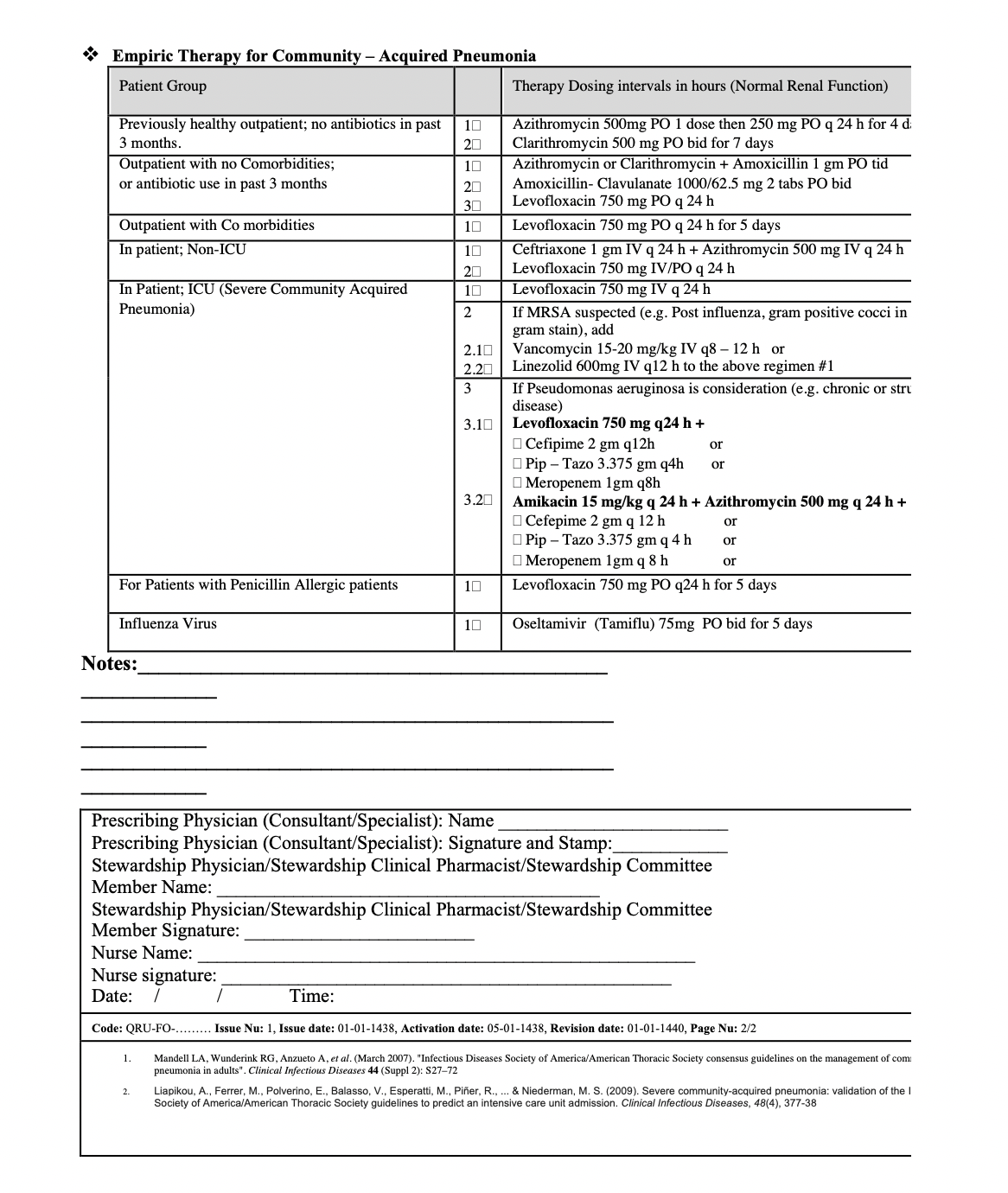
**

**
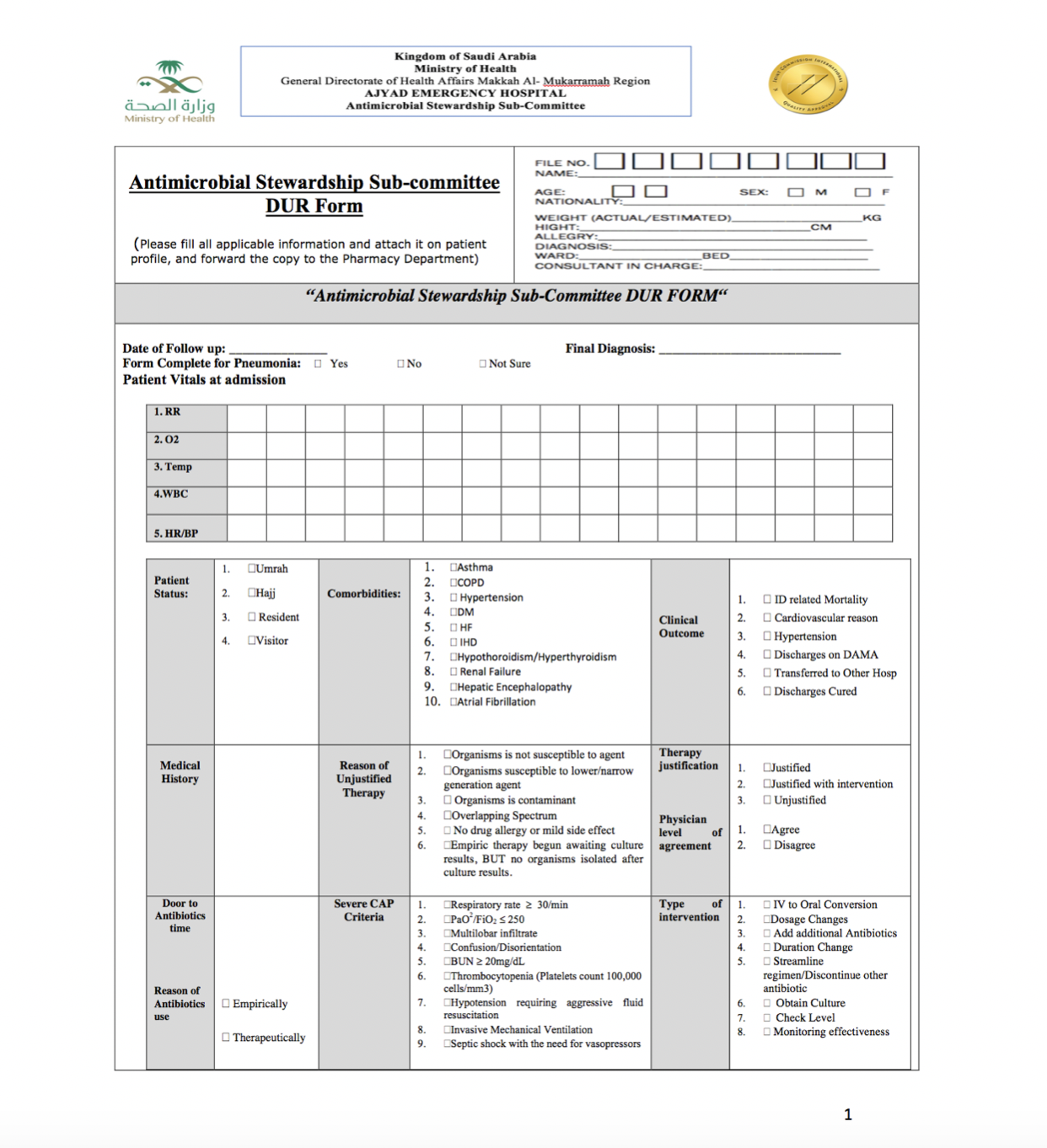
**

**List of Multidisciplinary Antimicrobial Stewardship Committee Members**

The committee included:

i Hospital director (head of the committee)

ii Medical director of the hospital (assistant head)

iii Clinical pharmacist with infectious disease training

iv ICU consultant/head of ICU with infectious disease training

v Head of pharmacy department

vi Clinical microbiologist

vii Head of quality department

viii Head of emergency department

ix Infectious disease physician at consultant level (visiting member)

**2.4.1 Detail of recommended ASP strategies used during study**

i Education and training of ICU specialists/residents regarding infectious disease guidelines and use of the hospital ASP toolkit in all shifts.

ii Education and training of hospital pharmacists regarding infectious disease guidelines and use of the hospital ASP toolkit in all shifts.

iii Clinical decision support system as predesigned antimicrobial prescribing forms according to the national antimicrobial prescribing guidelines for common infectious diseases. Physicians make decisions regarding antimicrobial choice according to the patient characteristics recorded during the admission process.

iv Antimicrobial prescribing policy to restrict use of broad-spectrum antibiotics to specific patients authorized by the hospital’s ASP team.

v IV to oral conversion if patients can tolerate oral antimicrobial intake without any variation in drug bioavailability.

vi Dose optimization using pharmacokinetic/pharmacodynamic modeling by clinical pharmacist. Vancomycin and aminoglycoside dosing recommended according to patient characteristics, for example, renal function and drug-specific pharmacokinetics. Therapeutic drug monitoring to be applied to antibiotics according to pharmacokinetic/pharmacodynamic monitoring criteria.

vii Renal adjustment of antibiotic dosing strategies (dose and dosing interval) performed according to renal function status (acute vs chronic renal failure) and stage (complete vs partial renal failure).

viii Regular audit and feedback to the intensivist by the ASP team during clinical rounds.
